# Targeting neuroinflammation to enhance recovery after brain injury

**DOI:** 10.1097/MS9.0000000000004642

**Published:** 2026-01-06

**Authors:** Muhammad Khizar, Muhammad Zaib, Meerab Babar, Mahnoor Fatima

**Affiliations:** a Faculty of Medicine, Georgian American University, Manifest Medical Research Co., Tbilisi, Georgia; b Faculty of Medicine, Rawalpindi Medical University, Rawalpindi, Pakistan; c Faculty of Medicine, Shaheed Ziaur Rahman Medical College, Rajshahi Medical University, Bogura, Bangladesh

**Keywords:** blood–brain barrier disruption, cytokine release, glial cells, neurodegeneration, neuroinflammation, traumatic brain injury (TBI)

## Abstract

Traumatic brain injury (TBI) continues to be a major global cause of morbidity and mortality, with neuroinflammation recognized as a central mechanism influencing both acute and long-term outcomes. Following the initial insult, activation of glial cells, cytokine release, and blood–brain barrier disruption drive a cascade of secondary injury. While these inflammatory processes contribute to debris clearance and neurorepair, excessive or prolonged activation leads to neuronal death and chronic neurodegeneration. Recent international research has focused on modulating these pathways to enhance recovery. In the United States and Germany, trials using stem cell derived exosomes and anti-cytokine biologics have shown neuroprotective potential. In China, novel compounds such as 3-monothiopomalidomide have demonstrated efficacy in reducing microglial activation and improving behavioral outcomes in experimental TBI. Integrating these pharmacological, cellular, and imaging innovations into clinical care could transform TBI management. This letter underscores the need for global collaboration, transparent data use, and biomarker-driven neuroinflammatory modulation to optimize adult brain injury recovery.

*Dear Editor,*

Traumatic brain injury (TBI) affects nearly 55 million people annually and remains a leading cause of adult disability worldwide^[1]^. Beyond the initial mechanical damage, secondary neuroinflammation has emerged as a key factor determining neurological recovery. The cascade of microglial activation, cytokine release, and oxidative stress disrupts neuronal connectivity and perpetuates neurodegeneration^[2]^. Though inflammation initially aids debris clearance and tissue repair, persistent activation leads to synaptic dysfunction and cognitive decline.

Advances in translational research across several countries highlight the therapeutic promise of targeting inflammation. In the United States and Germany, phase I/II trials investigating immunomodulatory agents, stem cell–derived exosomes, and anti-cytokine biologics have shown reduced neuroinflammatory markers and improved neurological function^[3]^. In China, preclinical studies using the thalidomide analog 3-monothiopomalidomide have demonstrated attenuation of glial hyperactivation and preservation of IL-10 signaling, resulting in improved behavioral outcomes after experimental TBI^[4]^. These findings reinforce the potential for pharmacological and cell-based immunomodulation to enhance neural recovery.

Clinically, the translation of these discoveries depends on precise monitoring and timing. Biomarker-guided imaging such as TSPO-PET for microglial activation and blood cytokine profiling are enabling real-time assessment of neuroinflammatory dynamics. Furthermore, nanocarrier drug delivery and regulatory T-cell therapy are being explored to selectively modulate neuroimmune responses. High-income countries like the USA and Germany are investing in large multicentric studies, while China’s rapidly expanding TBI research programs are driving innovation in molecular diagnostics. Together, these efforts exemplify how global cooperation can refine inflammation-targeted therapies for improved recovery outcomes.

In conclusion, neuroinflammation is both a pathological driver and a therapeutic target in adult brain injury. Emerging interventions that fine-tune inflammatory cascades, instead of suppressing them, offer a pathway toward more effective, individualized neurorehabilitation. Continued cross-border research, biomarker validation, and transparent AI-supported analytics will be essential to integrate these innovations safely into clinical practice. Our work is in line with the TITAN Guidelines on the need for transparency in AI use in health care^[5]^.

## Data Availability

Not applicable to this study.
